# Help for the London Poor

**Published:** 1889-11-16

**Authors:** 


### Help for the London Poor.

On Thursday afternoon, November 7, a meeting was held at The Lodge, Porchester-square, of the ministers of various denominations in a prescribed area of Kensington, to consider a scheme for dealing with poverty and distress amongst the poor of London.

Mr. BURDETT, who presided, said those present had already received copies of the scheme proposed.* It was decided, at a previous meeting, to take a limited area, and ask the ministers of all denominations within it to meet and decide what steps, if any, should be taken. The boundaries of this proposed district were as follows: the north was Golborne-road; the west, Ladbroke-grove-road and Ladbroke-grove; the south, Archer-street and Norfolk-terrace; the east, Ledbury-road and St. Luke's-road. In that district, the various religious agencies numbered fourteen. Ministers and those representing their agencies, who had been seen by the hon. secretary (Mr. D. Mackenzie), appeared willing to co-operate, and were invited to attend. Those who had been good enough to come were now asked to express their views, freely and frankly. The wish was that, if they agreed to organise this small district of Kensington as a nucleus for a greater work, they should form themselves into a pro-

* *See* THE HOSPITAL, August 10th, 1889, Vol. VI., page 299 *et seq*

visional committee, which would decide what steps should be taken with a view to having a further meeting of the representatives of the various congregations, and trying to bring into the work all who were concerned in the relief of the poor within the area. Mr. Mackenzie had found the ministers willing to engage in a scheme which promised some hope of finality. Many necessitous persons were known only to the workers, persons, that is, who wanted friends, having none at present. They would have the means of knowing about such persons, and feeling that they were being looked after in the best way. From what he had seen in Paddington, and in Boston, U.S., he felt that a scheme on the lines proposed would be successful. If they could take advantage of the division of London into Parliamentary units, and endeavour to awaken in the various districts local life, such as they found in provincial towns, the question of the great mass of unrelieved distress could be really dealt with. They had enough money in the aggregate to more than meet the distress, but owing to special circumstances, it was so scattered, and to a certain extent wasted, that there was often the feeling that more relief was needed than there was money to pay for. He would not delay them now, but ask the earnest men who had come together what steps could be taken, or whether it was desirable to do anything. He was never an advocate for doing nothing. They ought to work from a central basis, but they must deal with the individuals according to the district in which they resided.

The Rev. Dr. Sinclair Paterson (Presbyterian) said he was obliged to leave, but would gladly co-operate in any decision arrived at.

The Rev. W. Roberts (Horbury Chapel) said he was present as a resident in the district, though his work lay outside it. He felt strongly that the money employed for helping the poor in London was more than enough, but it was found they had the greatest practical difficulties in helping others. The poor, in some cases, got money from many of the agencies which existed, and those not really in want might get a large share. If they could detach all this money given for the poor from all their churches, so that there should be a general organisation, on the principle of Christian citizenship, it would be the most effective way of doing it. So long as particular churches in particular districts acted as at present, over-lapping would result. He had long been waiting for some plan to meet the difficulty.

The Rev. J. Tuckwell (Westbourne-grove Baptist Chapel) called attention to what he found to be the custom of the Paddington Guardians. They considered 10s. a week a maximum sum for meeting actual necessities. He had suggested that with this rule it was clear the guardians could decide the maximum, and therefore could put it at 12s. if they wished, instead of 10s. The chairman of the board had admitted that this was so. The guardians, then, must give this 10s. a week to a poor family, and if they discovered that the family had 2s. a week from any other source, they would reduce their grant to 8s. The case had been instanced of a poor woman who, by the work and care of her husband, had 6s. a week coming in, and the guardians could only give 4s. to make up 10s. How were they—concerned with voluntary charity—to meet that difficulty?

Canon Trench said there was an important letter in the *Times*, of October 14, from Sir Edmund Currie, showing from his experience that it was quite possible for a voluntary organisation to work in conjunction with the board of guardians. He (Sir Edmund Currie) thought that was the only efficient way of doing it. The thing was done fairly successfully some years ago, and in view of winter needs Sir E. Currie thought that was what should be done.

The Chairman said the letter referred to dealt with Mr. Tuckwell's question. If a voluntary organisation had adequate knowledge of all cases, the guardians would be most glad to have its help. It was immensely difficult to get people to combine, but if they started with an earnest desire to do good work in the best way, it was only a question of getting over the initial shyness, and making an experiment which would produce results so satisfactory that everyone would wonder it had not been done before. In Paddington, the Vicar of St. Paul's, and the Rev. Dr. Clifford, and the Roman Catholic clergyman had worked together now for some time, and had absolutely succeeded in getting in touch with the population in their district. Dr. Clifford had told him on Saturday last they were now organising a labour bureau, and two or three of the best men would take charge of it. They hoped to get good results, though well knowing it was a difficult undertaking. The proposal simply meant that every pastor in the district and his workers should form themselves into a conference, and the workers would thus have the right to come before this conference with particular cases, about which they knew everything, and thus have the benefit of the wisdom and experience of all in dealing with them. This would not interfere with existing agencies, but simply supplement the knowledge of all and increase the power of doing good. They would have to deal with the question of supplementary funds, and how they were to deal, if at all, with existing funds. Then, how far present funds should be utilised through the conference. People would give when they believed the conference knew what it was about, and when they approved the plan on which it was working. As to the suggestion that people would come for relief from outside, they would relieve no one not resident in the district for six months. A simple rule like that would moderate the evil, and they could soon devise means which would effectually check it. They could not stop individual gifts to particular people, but the conference would exercise a moral influence, and people would soon come to find the best way to give help was to give it through their own clergyman, or send it through him. Of course, if they could get every householder to look after one poor famlly, it would do an immense amount of good. As to a central organisation, the object was to concentrate knowledge, so that each worker might know what was being done in particular cases. It would not be a foreign or outside body interfering with workers. The desire was that they should be able to know about their own immediate neighbours, and to ensure that no poor man or woman should suffer unnecessarily. He believed the Poor Laws required improvement, and the Government was considering the matter. An organisation like the one proposed would help to show what changes were desirable.

Canon Trench said there was one way in which the organisation would be of special use. The guardians had one method and the Charity Organisation Society another of helping the poor, but in both cases application had to be made to these agencies for relief by the people themselves. Very often people from various causes were indisposed to make application. Some of the most worthy people were the most indisposed to make it. The less worthy were the best beggars. Now an organisation like this would search out the poor and help them. It would greatly encourage poor people to know how they could be helped. By thus gaining access to the poor, they could reach cases which neither the Charity Organisation Society nor the Poor Law Guardians were ever asked to touch. From knowledge of the facts, they would then decide what was the best method of relief in each individual case.

In answer to a question, the Chairman said his own idea was that they should not, if possible, give relief in money, but provide work, or help in some other form. In Paddington they had given £1,500 away, and only £50 of that sum, he believed, in money; the rest for services rendered. They could not change the habits of people all at once, or bring them on the instant, for example, to their own views of what made a home comfortable. The main principle of the scheme was love for their fellow-men, and a desire to encourage them to help themselves.

The Rev. J. Stevens (Congregationalist) and Mr. Donald Mackenzie (hon. secretary) also contributed to the discussion, and Father Sylvester suggested opening communications with Mr. S. Lawrance, the president of the Society of St. Vincent de Paul in the district prescribed. The Chairman said he would gladly ask Mr. Lawrence for his assistance.

Finally, on the motion of the Rev. J. Stevens, seconded by Rev. Canon Trench, it was resolved: "That the gentlemen who were invited to attend this meeting be appointed a provisional council, and that the Rev. Canon Trench, Rev. Father Sylvester, Rev. Father Kirk, Rev. W. Roberts, Rev. J. Tuckwell, Rev. J. Stevens, Rev. Dr. Paterson, Rev. C. J. Ward, Rev. R. H. Roberts, Rev. F. White, and Mr. S. Collett, with Mr. Donald Mackenzie as hon. secretary, be requested to act as an executive committee, with a view of giving the scheme a fair trial in the district selected."

